# Apomixis for no bacteria-induced thelytoky in *Diglyphus wani* (Hymenoptera: Eulophidae)

**DOI:** 10.3389/fgene.2022.1061100

**Published:** 2023-01-23

**Authors:** Sujie Du, Fuyu Ye, Shiyun Xu, Yongxuan Liang, Fanghao Wan, Jianyang Guo, Wanxue Liu

**Affiliations:** ^1^ State Key Laboratory for Biology of Plant Diseases and Insect Pests, Institute of Plant Protection, Chinese Academy of Agricultural Sciences, Beijing, China; ^2^ College of Life Sciences, Hunan Normal University, Changsha, Hunan, China

**Keywords:** cytological mechanism, karyotype, apomictic thelytoky, microsatellite, heterozygosity

## Abstract

In Hymenoptera species, the reproductive mode is usually arrhenotoky, where haploid males arise from unfertilized eggs and diploid females from fertilized eggs. In addition, a few species reproduce by thelytoky, where diploid females arise from unfertilized eggs. Diploid females can be derived through various cytological mechanisms in thelytokous Hymenoptera species. Hitherto, these mechanisms were revealed mainly in endosymbiont-induced thelytokous Hymenoptera species. In contrast, thelytokous Hymenoptera species in which a reproductive manipulator has not been verified or several common endosymbionts have been excluded were paid less attention in their cytological mechanisms, for instance, *Diglyphus wani* (Hymenoptera: Eulophidae). Here, we investigated the cytological mechanism of *D*. *wani* using cytological methods and genetic markers. Our observations indicated that the diploid karyotypes of two strains of *D*. *wani* consist of four pairs of relatively large metacentric chromosomes and one pair of short submetacentric chromosomes (2*n* = 10). The arrhenotokous strains could complete normal meiosis, whereas the thelytokous strain lacked meiosis and did not expulse any polar bodies. This reproductive type of lacking meiosis is classified as apomictic thelytoky. Moreover, a total of 636 microsatellite sequences were obtained from thelytokous *D*. *wani*, dominated by dinucleotide repeats. Genetic markers results showed all three generations of offspring from thelytokous strain maintained the same genotype as their parents. Our results revealed that *D*. *wani* is the first eulophid parasitoid wasp in Hymenoptera whose thelytoky was not induced by bacteria to form an apomictic thelytoky. These findings provide a baseline for future inner molecular genetic studies of ameiotic thelytoky.

## 1 Introduction

The most common form of reproduction in Hymenoptera with haplodiploid sex determination is arrhenotokous, in which haploid males develop from unfertilized eggs and diploid females develop from fertilized eggs (Luck et al., 1993; Heimpel and de Boer, 2008). However, more than 500 Hymenoptera species have been documented as thelytokous (unfertilized eggs which generally develop into diploid females) (van der Kooi et al., 2017; Du et al., 2022).

**FIGURE 1:**
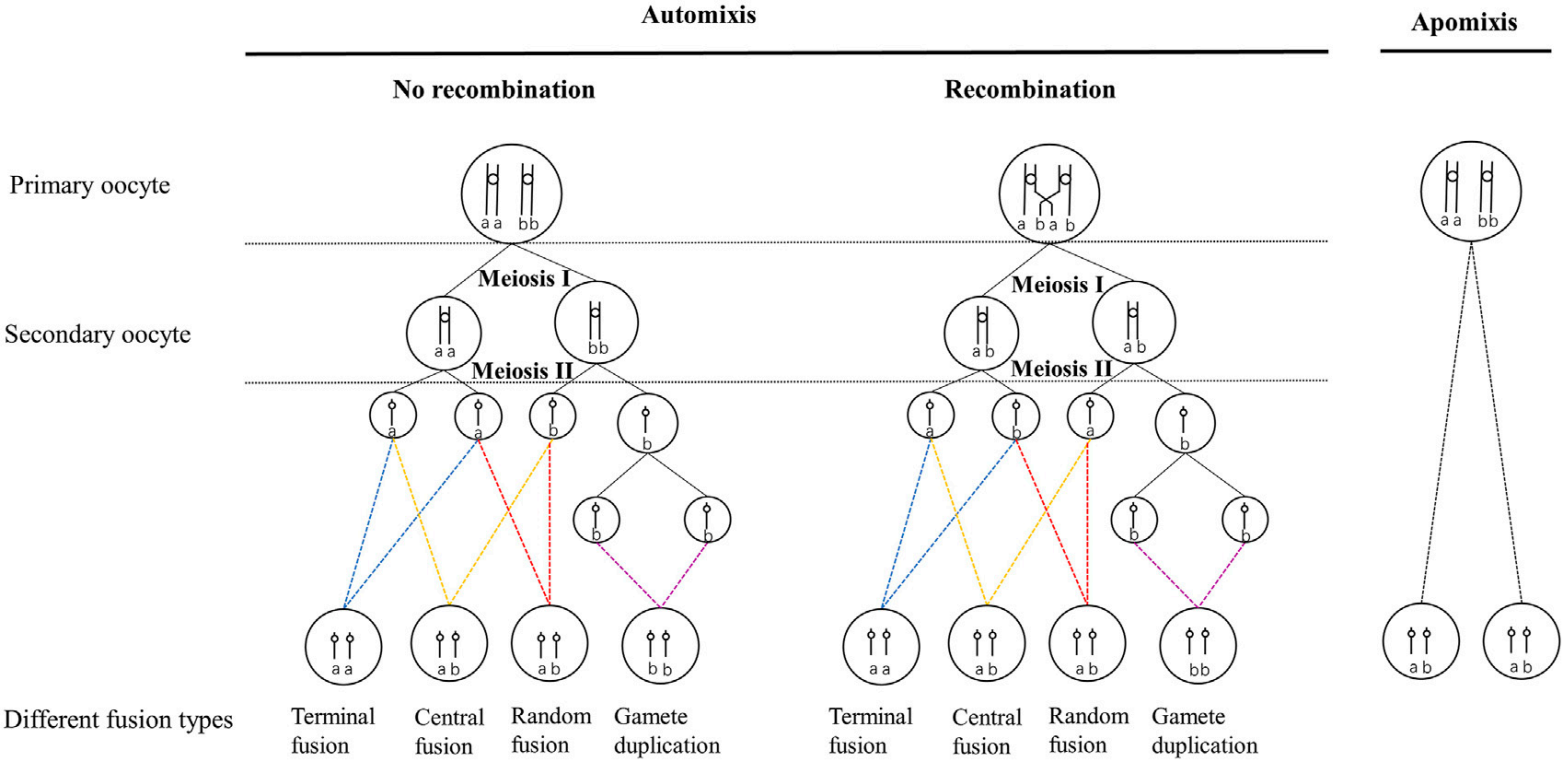
Different cytological mechanisms of automictic thelytoky during meiosis and their genetic effects on progeny at a locus in the event of and without recombination. The circle represents the nuclei. Vertical lines indicate chromatids, small circles connecting vertical lines indicate centromeres, and the lowercase letters indicate alleles at specific loci. The four different colored dashed lines point to four different cell fusion processes of automixis. The parent is heterozygous, indicated by ab. Homozygous progeny was indicated by aa or bb.

The production of diploid females from unfertilized eggs can be achieved through automixis and apomixis (Suomalainen et al., 1987; Gottlieb et al., 2002; Pearcy et al., 2006; 2011b). Under automixis, normal meiosis can be completed (Suomalainen et al., 1987; Archetti, 2010), and diploid recovery is usually accomplished by terminal fusion, central fusion, gamete duplication, or random fusion (Suomalainen et al., 1987; Pearcy et al., 2006) (Figure 1). However, apomixis lacks a meiotic process or meiosis in which one division is suppressed (Suomalainen et al., 1987; Archetti, 2010), and diploid female offspring are clones of their mothers due to the lack of genetic recombination (Suomalainen et al., 1987; Pearcy et al., 2006; 2011b).

Automictic thelytoky is a common cytological mechanism in Hymenoptera, including Chalcidoidea, Ichneumonoidea, and Cynipoidea (Suomalainen et al., 1987; Sanderson 1988; Beukeboom and Pijnacker, 2000; Belshaw and Quicke, 2003; Vavre et al., 2004; Adachi-Hagimori et al., 2008; Mateo Leach et al., 2009; Tsutsui et al., 2014). Under automixis, gamete duplication was reported more frequently than under the other three cytological fusion modes (Ma and Schwander, 2017). In addition, gamete duplication has been investigated in thelytokous parasitoids infected with bacteria such as *Wolbachia*, *Rickettsia,* and *Cardidium* (Stouthamer and Kazmer 1994; Gottlieb and Zchori-Fein, 2001; Pannebakker et al., 2004; Giorgini et al., 2007; Simonato et al., 2019).

In contrast, apomixis is uncommon in Hymenoptera (Van Wilgenburg et al., 2006; Rabeling & Kronauer, 2013), and it has been reported in some social ants which were found to be functionally mitotic, such as *Paratrechina longicornis* (Pearcy et al., 2011a) and *Cataglyphis hispanica* (Leniaud et al., 2012). To our knowledge, apomixis has been found only in seven thelytokous Hymenoptera species except eusocial species. These seven apomictic thelytokous Hymenoptera species include: three phytophagous sawfly pests [e.g., *Pachyprotasis youngiae* (Naito and Inomata, 2006), *Strongylogaster macula* (Peacock and Sanderson, 1939), *Nematus oligospilus* (Caron et al., 2013)], one gall wasp (*Neuroterus baccarum*) (Doncaster, 1916; Dodds, 1939), and three parasitoids [e.g., *Trichogramma cacaeciae* (Vavre et al., 2004), *Neochrysocharis formosa* (Adachi-Hagimori et al., 2008), and *Meteorus pulchricornis* (Tsutsui et al., 2014)]. However, the apomixis of these seven species was produced under different cytological processes. For example, thelytokous *N. formosa* and *T. cacaeciae* did not proceed to meiotic recombination and reduction, whereas only undergone a single equational division followed by the expulsion of a single polar body in the meiotic process (Vavre et al., 2004; Adachi-Hagimori et al., 2008). Nevertheless, meiosis was completely absent in *M. pulchricornis* (Tsutsui et al., 2014). On the whole, the cytological mechanisms of many thelytokous female parasitoids have not been fully elucidated.

The cytological mechanisms of thelytoky could be investigated through genetic markers in addition to cytological observations (Suomalainen et al., 1987; Pearcy et al., 2006; 2011b; Wachi et al., 2021). Cytological observation of newly laid eggs is a straightforward method to determine the cytological mechanism at present. The genetic effects obtained by the offspring from the parents change due to the different fusion methods under automixis and apomixis (Pearcy et al., 2006; 2011b). Genetic markers (alleles) have also been used to investigate cytological mechanisms (Adachi-Hagimori et al., 2008; Tsutsui et al., 2014). For parents with a heterozygous locus, the probability of a heterozygous locus becoming homozygous for each cytogenetic mechanism is distinct (Pearcy et al., 2006; Pearcy et al., 2011b). The theoretical rate of transition to homozygosity of a heterozygous locus during parthenogenesis is expected to be 0 in apomixis, and 0–1/3 in automixis with central fusion, 1/3–1 with terminal fusion, and 1 with gamete duplication (Pearcy et al., 2006; Pearcy et al., 2011b). Thus, the cytological mechanism of thelytoky of a species could be determined by the homozygous rate of offspring produced by heterozygous parents (Pearcy et al., 2006). Application of the theories proposed by Pearcy et al. (2006); Pearcy et al. (2011b) has been observed in many species, including Hymenoptera (Vavre et al., 2004; Adachi-Hagimori et al., 2008; Tsutsui et al., 2014; Smith et al., 2019), Phasmatodea (Alavi et al., 2018), and Blattodea (Tanaka and Daimon, 2019).

Here, we focused on the dominant natural enemy of agromyzid leafminers, *Diglyphus wani*, which has been identified as thelytokous and arrhenotokous (Du et al., 2021). *Diglyphus wani* was not infected with *Wolbachia*, *Rickettsia*, *Cardinium,* or any other known bacterial reproductive manipulator (Du et al., 2022). This study aimed to explore the cytological process of restoring ploidy in thelytokous *D*. *wani* using cytological observation and microsatellite genetic markers. Our study could provide a theoretical basis for further research into the genetics of *D*. *wani*.

## 2 Materials and methods

### 2.1 Insect cultures

Thelytokous and arrhenotokous *D. wani* were originally collected from Xining, Qinghai, China (36°39′N, 101°45′E) in 2015 and Kunming, Yunnan, China (24°53′N, 102°47′E), respectively. Fresh *Phaseolus vulgaris leaves* were provided as plant hosts for *Liriomyza sativae* in the laboratory. Leaves containing 2nd to 3rd instar larvae of *L. sativae* were used as hosts for parasitoids. The leafminers and parasitoid colonies were stably reared for multiple generations at 14L: 10D photoperiod and 25°C ± 1°C.

### 2.2 Karyotype analysis

Metaphase chromosomes were obtained from 4-day-old larvae of two strains of *D*. *wani* processed using the method described by Imai et al. (1977), with minor modifications. Fifteen larvae each of thelytokous and arrhenotokous strains were collected. These larvae were nicked with a small cut between the head and thorax in 1.0 ml 0.1% colchicine in Shen solution. Metaphase plates were examined using an Olympus BX61 microscope (Tokyo, Japan). Chromosomes were imaged and measured with an Olympus DP72 camera (Japan) using Olympus CellSens Dimension 1.5 software. To prepare illustrations, the resulting images were arranged and enhanced using Adobe Photoshop 22.0 software. The mean and standard error of the chromosome length were calculated using Microsoft Excel 2016. The chromosomes were classified according to the guidelines described by Levan et al. (1964).

### 2.3 Cytogenetic observation

To observe the meiotic and mitotic stages in newly laid eggs, 60 female adults of each strain of *D. wani* were provided with sufficient 2nd to 3rd instar larvae for egg laying. The leaves containing *L*. *sativae* larvae were kept fresh in round Petri dishes with 10 ml water agar (1%). The parasitoids were allowed to oviposition for 30 min and then removed. Newly laid eggs on or near the surface of the host larvae were kept for 30 min at time intervals from 0 to 120 min after oviposition. Eggs were sequentially fixed on a microscope slide by fixatives as follows: 1) distilled water: absolute ethanol: glacial acetic acid at 4:3:3, 2) absolute ethanol: glacial acetic acid at 1:1, and 3) glacial acetic acid. After air-drying, fixed eggs were stained with 4′,6-diamidino-2-phenylindole (DAPI) (Solarbio Science & Technology Co., Ltd. Beijing) and covered with an antifluorescent quenching agent (Solarbio Science & Technology Co., Ltd. Beijing) using cover glasses. The imaging system used was the same as that used for karyotype analysis, except that it was equipped with epifluorescence. Over 100 eggs were examined for the two strains of *D. wani*.

### 2.4 Construction of a microsatellite library

#### 2.4.1 DNA extraction for microsatellite library

We randomly collected six adult female thelytokous *D*. *wani* reared in laboratory colonies. These specimens were not allowed to eat and were collected within 12 h of emergence. Then, they were rapidly frozen in liquid nitrogen and stored at −80°C for DNA extraction. DNA was extracted using the salting-out DNA extraction method (Patwary et al., 1994).

#### 2.4.2 Microsatellite enrichment

Parasitoid genomic sequences were fragmented using a Covaris Ultrasonic DNA Fragmenter (S220; United States). Libraries with fragment lengths of approximately 500 bp were prepared using the NEB Next^®^ Ultra™ DNA Library Prep Kit for Illumina^®^ (E7370, New England Biolabs). Briefly, DNA fragments were end-repaired and ligated. The ligated products were sorted and purified using biotin-coated magnetic beads (Hieff NGS DNA Selection Beads, Yeasen Biotechnology, Shanghai, Co., Ltd.). Two pair primers (index primer/i7 primer and universal PCR primer/i5 primer) that come with the DNA Library Prep Kit were used for PCR amplification. The amplification was performed on the purified junctional products and the PCR-amplified products were purified and enriched. PCR amplification products of the eluted fragments were ligated with the cloning vector and transferred into *E*. *coli* receptor cells to construct a cloning library of enriched microsatellites.

#### 2.4.3 Screening of sequencing and design of microsatellite primers

The enriched fragments were sequenced using Illumina MiSeq (Illumina, San Diego, California, USA) at Shanghai Sangon Biotechnologies using a 2 × 300-bp paired-end sequencing strategy. Contigs containing microsatellite repeats were identified by the MicroSAtellite Identification Tool (MISA) (Thiel et al., 2003). SSR Hunter software 1.3 (Li and Wan, 2005) was used to calculate the number of nucleotide repeats in the microsatellite sequence, with default parameters for mono-, di-, tri-, tetra-, penta-, and hexanucleotide repeat types, with a minimum of 10, 6, 5, 5, 4, and 4 repeats, respectively. We also calculated the number of perfect, compound, and imperfect microsatellites. The categories of perfect, compound, and imperfect microsatellites were carried out according to the standard proposed by Weber (1990).

Forty-two contigs were randomly selected to develop polymorphic markers (Supplementary Table S1; Table 3). We designed a set of forward and reverse primers using Primer Premier ver. 5.0 (Clarke and Gorley 2001). The designed primers were screened to determine whether the microsatellite sequence could be successfully amplified using template DNA extracted from eight thelytokous *D*. *wani* adult individuals from the laboratory colony using the rapid method of De Barro and Driver (1997). A single adult individual was thoroughly grinded using a sterilized 200-µL pipet tip whereas pointy end of tips was fused by the alcohol lamp. Then the individual parasitoid adult was incubated in 30-µL lysis buffer (50 mM KCl, 10 mM Tris pH 8.4, 0.45% Tween 20, 0.2% gelatin, 0.45% Nonidet P-40, 60 mg/ml proteinase K). The incubation program was 65°C 30 min, 25°C 2 min, and 95°C 5 min. Then the 5′end of forward primers were tagged with a fluorescent FAM dye for further analysis. Finally, 10 genetic markers were selected to investigate the genotypes of thelytokous *D*. *wani*. The principles for selecting these 10 genetic markers are as follows: 1) the primer designed from 42 contigs can successfully amplify the target electrophoresis band without impurity; 2) The peak map is not cluttered when genotyping; 3) to choose potential markers for subsequent validation of allele isolation, the genotype of thelytokous *D*. *wani* in the laboratory should be heterozygous in at least one genetic marker.

### 2.5 Amplification of microsatellite loci from thelytokous *Diglyphus wani* in the field

Single parasitoid DNA was rapidly extracted according to the protocol developed by De Barro and Driver (1997). Amplification of microsatellite fragments was carried out as follows: 0.1 μL *Taq* enzyme (2.5 UμL^−1^), 0.2 μL dNTPs (2.5 Mm), 2.5 μL 10× buffer (containing Mg^2+^), 0.2 μL forward primer, 0.2 μL reverse primer, 1 μL DNA template, and ddH_2_O to 25 μL. The PCR program were set as: 95°C for 3 min, followed by 35 cycles of denaturation at 95°C for 20 s, annealing for 20 s, extension at 72°C for 25 s, and a single cycle of final extension at 72°C for 5 min. The PCR instrument used was an ABI thermal cycler (Veriti Applied Biosystems 9902, Singapore). Genotyping was performed using Sangon Biotechnologies (Shanghai, China).

To investigate polymorphic loci and the degree of genotype heterozygosity based on microsatellite primers, 20 field-collected individuals from Tibet, Xinjiang, Ningxia, and Qinghai, and four laboratory individuals were used for the test (Table 1). Microsatellite profiles of the field specimens were examined using GeneMapper version 4.0, and the alleles were scored manually. CERVUS ver. 3.0.7 was used to analyse the number of alleles, observed heterozygosity (HO), expected heterozygosity (HE), and the polymorphic information content (PIC) of the microsatellite loci (Kalinowski et al., 2007).

**TABLE 1:** Populations of thelytokous *D. wani* for testing polymorphisms of microsatellite loci.

Date	Province	City	Coordinated system	Host plant	Host	Individuals
201807	Tibet	Lhasa	29°38′N, 91°02′E	*Pisum sativum*	*Chromatomyia horticola*	5
201806	Ningxia	Guyuan	36°08′N, 106°14′E	*Brassica juncea*	*Chromatomyia horticola*	5
201806	XinJiang	Kizilsu Kirghiz	39°56′N, 75°32′E	*Clematis florida*	*Phytomyza* sp	4
201807	Qinghai	DeLingHa	37°22′N, 97°22′E	*Senecio vulgaris*	*Chromatomyia horticola*	6
201507	Qinghai	Xining	36°39′N, 101°36′E	*Phaseolus vulgaris*	*Liriomyza sativae*	4

### 2.6 Patterns of allele segregation

To investigate the inheritance patterns of allele segregation, DWTH 119, DWTH 366, DWTH 178, and DWTH 340 locis were selected as candidate gene markers. In addition, to investigate whether these four loci segregated and originated from the nuclear region, we detected allele segregation between parents and offspring of heterozygous arrhenotokous *D*. *wani*. Segregation of these four alleles occurred in the process of producing male offspring from parent females of arrhenotokous *D*. *wani*, because males carried only one allele of the heterozygous locus. Eight females from the laboratory colonies were allowed to oviposit on the 2nd to 3rd instar larvae of *L*. *sativae* for their entire life. Single individuals were used for DNA extraction, following the method described by De Barro and Driver (1997). For each allele, we analyzed the proportion of homozygous genotypes produced by heterozygous mothers, and this proportion is denoted by observed value *R*. We compared *R* values with theoretical expectations *r* for different cytological mechanisms of thelytoky: apomixis (*r* = 0), automixis with gamete duplication (*r* = 1), terminal fusion (*r* = 1/3–1), fusion of two products of the first meiotic division, here referred to as random fusion (*r* = 1/3), and central fusion (*r* = 0–1/3). These comparisons are based on those reported by Pearcy et al. (2006). The value closest to the observed *R* was utilized for the test when *r* was within the range of values. We utilized Fisher’s exact tests to evaluate the likelihood of conformity between theoretical and expected values, and significant values with a 95% confidence interval (95% CI) were employed. Statistical analysis was performed using SPSS 20.0 software.

## 3 Results

### 3.1 Karyotype analysis

**FIGURE 2:**
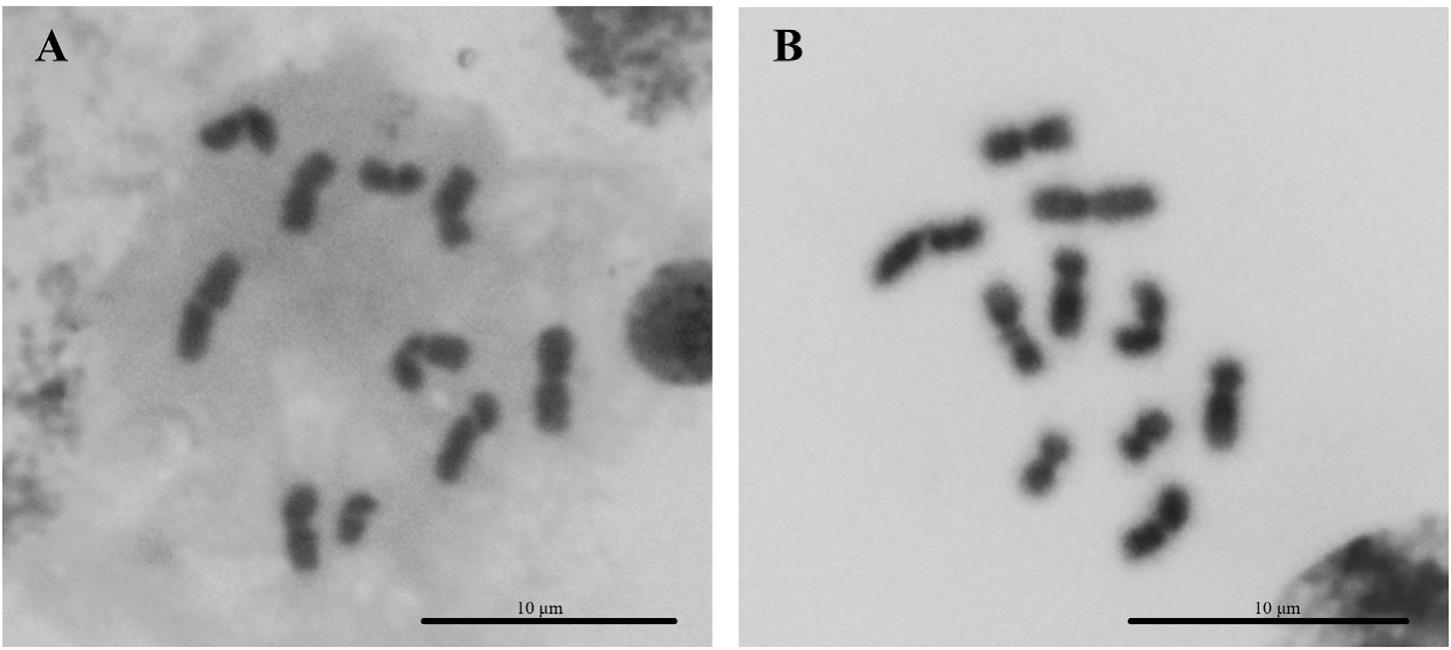
Karyotypes of two strains of *Diglyphus wani*. **(A)** Thelytokous female (2*n* = 10) **(B)** arrhenotokous female (2*n* = 10).

The overall metaphase chromosomal morphology of both arrhenotokous and thelytokous *D*. *wani* was similar (Figure 2). Both had diploid female karyotypes comprising four pairs of relatively large metacentric chromosomes and one pair of submetacentric chromosomes (2*n* = 10) (Figures 2A, B; Table 2).

**TABLE 2:** Karyotypic parameters of females of the two strains of *D. wani*: Mean ± Standard deviation. Chromosomes are classified into metacentric (M), submetacentric (SM), and acrocentric (A) according to the CI value of Levan et al. (1964).

Strain	Chromosome pair	Relative length (RL)	Centromeric index (CI)	Classification
Thelytokous *D*. *wani*	1	27.00 ± 1.34	46.54 ± 2.31	M
	2	21.74 ± 1.10	47.06 ± 2.00	M
	3	20.41 ± 0.49	34.76 ± 2.14	SM
	4	18.46 ± 1.08	45.76 ± 2.06	M
	5	12.38 ± 0.99	43.05 ± 2.13	M
Arrhenotokous *D*. *wani*	1	25.38 ± 1.47	46.96 ± 1.95	M
	2	21.55 ± 0.71	47.29 ± 1.32	M
	3	20.14 ± 0.73	35.42 ± 1.27	SM
	4	19.30 ± 0.72	45.82 ± 2.47	M
	5	13.63 ± 0.94	45.41 ± 3.45	M

### 3.2 Cytogenetic analysis

#### 3.2.1 Observation of the fertilization process of the arrhenotokous strain

**FIGURE 3:**
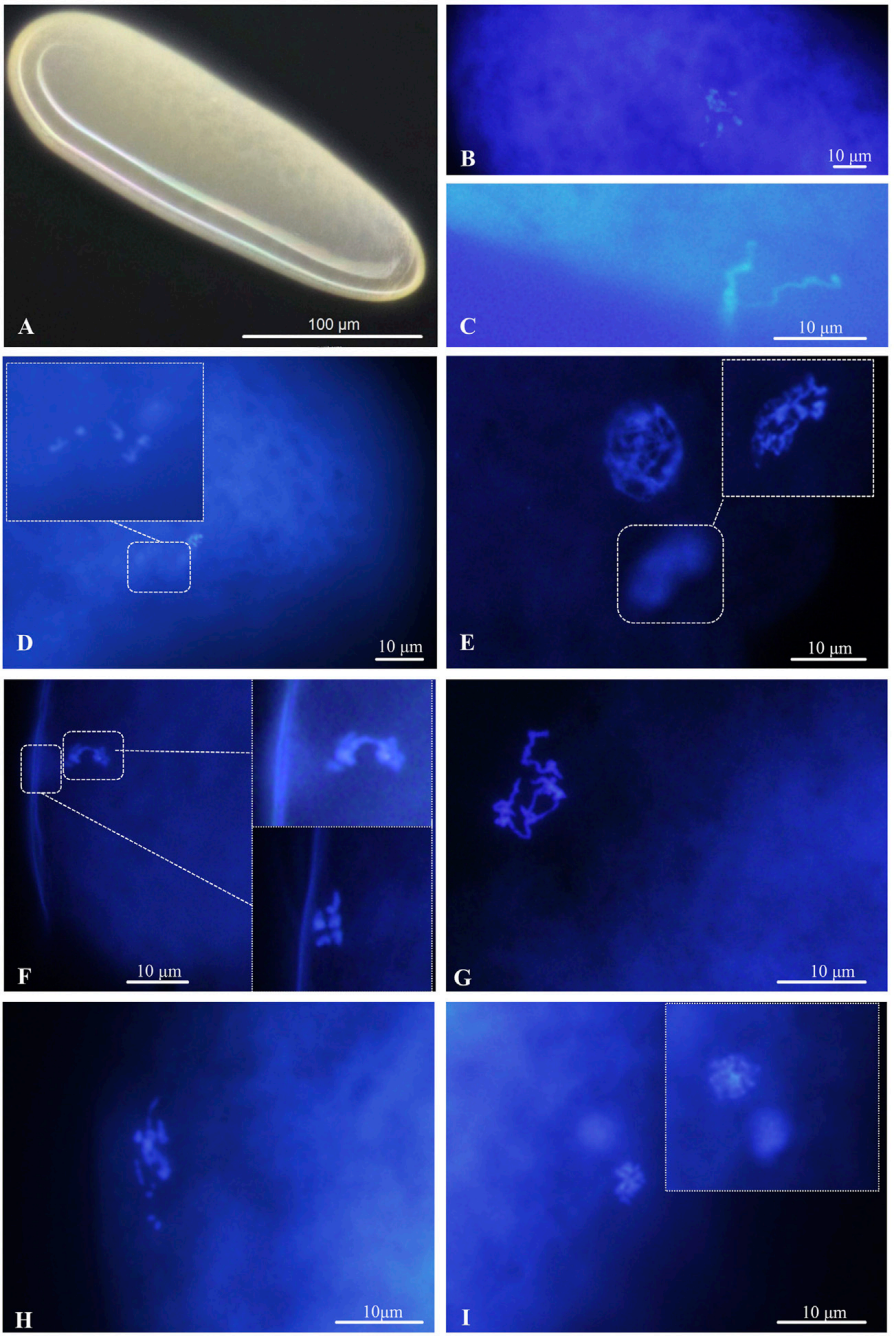
Chromosomes in the deposited eggs of the arrhenotokous strain. **(A)** Egg, creamy white or yellowish white, anterior at the top; **(B)** First metaphase of meiosis after oviposition; **(C)** sperm, found in freshly laid and sectioned eggs; **(D)** first anaphase of meiosis; **(E)** prophase of second meiosis; **(F)** second anaphase of meiosis and the first polar body with 5 chromosomes delayed at metaphase; **(G)** despiralized spermatozoon **(H)** the end of fertilization of female and male pronuclei **(I)** diploid telophase of first somatic mitosis.

The newly deposited eggs were elongate-oval, mostly creamy white or yellowish-white, and broader at the anterior end (Figure 3A). Newly laid eggs were approximately 240.69 µm in length, 77.46 µm in width, and sometimes filled with yolk (*n* = 11) (Figure 3A). For cytological analysis, arrhenotokous females deposited eggs in the host in the first meiotic metaphase. The number of chromosomes in the first metaphase occurred in the anterior part of the eggs; they could not be counted because the bivalents clustered very closely together (Figure 3B). At this point, two sperm attached to the egg surface were observed, and the sperm head and flagellum were clearly visible (Figure 3C). The bivalents remained in the first metaphase until 30 min after oviposition, when a set with the haploid number of five chromosomes could be seen moving towards the periphery, evidencing a reduction division (Figure 3D). The chromosomes were in the first telophase after 1 hour and demonstrated despiralization from late anaphase onwards (Figure 3D).

The second meiosis began in the next 30 min, forming a large interphase nucleus and a small interphase polar body nucleus (Figure 3E). After another 30 min, the second set was observed to divide parallel to the periphery and resulted in two groups of five chromosomes, while the first polar body with five chromosomes was delayed at metaphase (Figure 3F). At the end of meiosis, sperm in interphase were ready for fertilization (Figure 3G). Soon after, we only observed the scene at the end of fertilization, and chromosome numbers returned to those of pre-meiosis (Figure 3H). Once the eggs were fertilized, the first mitotic division began. Normally, the first division of diploid somatic cells occurs 2-3 h after egg laying (Figure 3I).

#### 3.2.2 Diploidization of thelytokous strain

**FIGURE 4:**
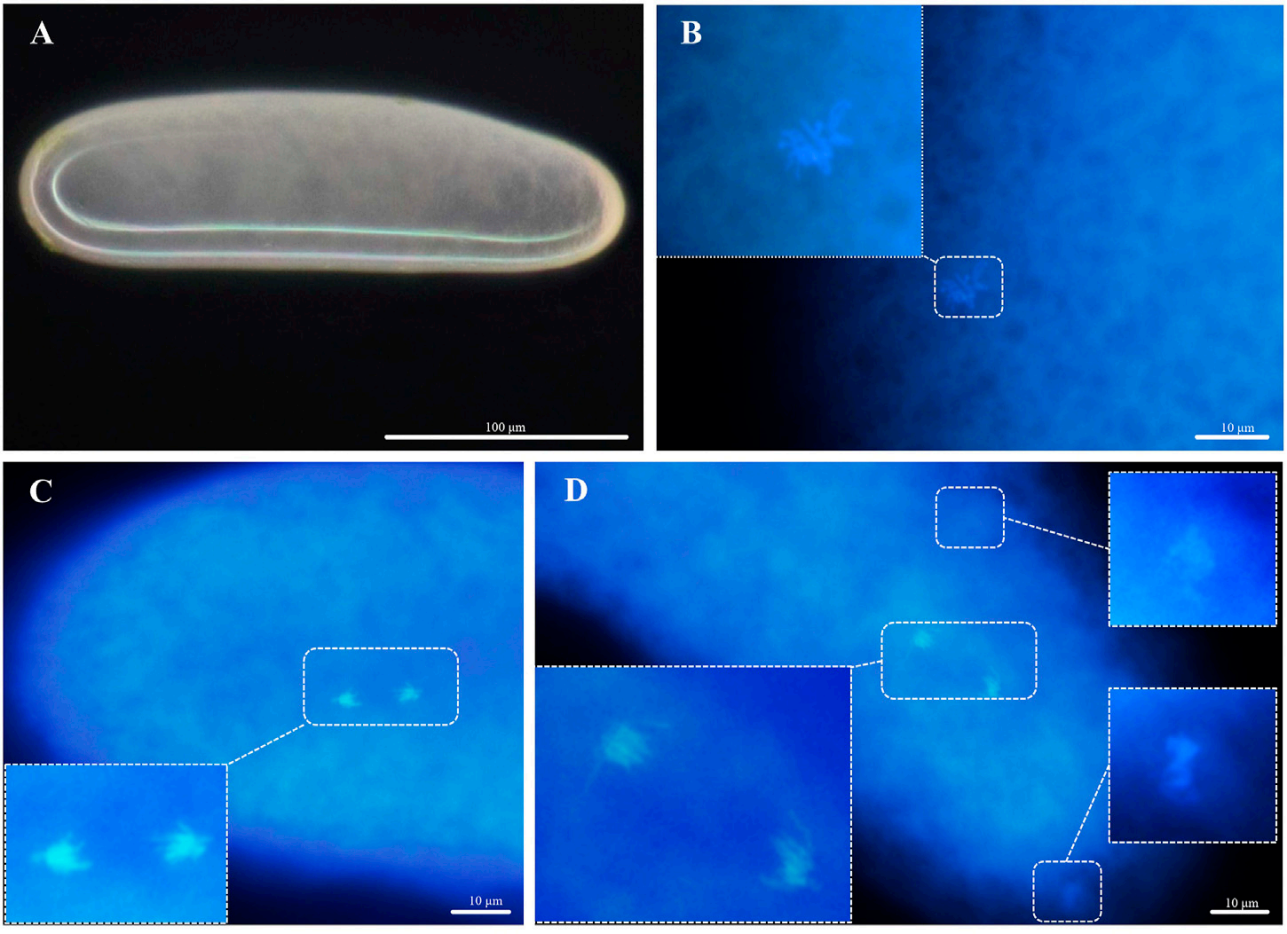
Chromosomes in the deposited eggs of the thelytokous strain **(A)** egg, creamy white, anterior at the top; **(B)** first metaphase **(C)** first anaphase; **(D)** second telophase.

Unlike the arrhenoyokous strain, newly laid eggs of the thelytokous strain were usually creamy white, approximately 256.62 µm in length, 75.12 µm in width, and generally lacking yolk (*n* = 18) (Figure 4A). Cytological analysis of the thelytokous strain showed that freshly laid eggs were in metaphase of division (Figure 4B). Similar to the arrhenotokous strain, the chromosomes at this stage were arranged in a bewildering manner, which prevented their number from being assessed in the thelytokous strain. After 30 min, we observed anaphase of parallel division, resulting in the formation of two sets of products with approximately 10 chromosomes (Figure 4C). The second anaphase of parallel division was also observed on approximately 10 chromosomes (Figure 4D). These divisions were not reduced, indicating that the division underway at this time was mitosis rather than meiosis. In subsequent observations, up to 2 h, the divisions were still not reduced, and the division process was the same.

### 3.3 Microsatellite characterization and primers combination

**FIGURE 5:**
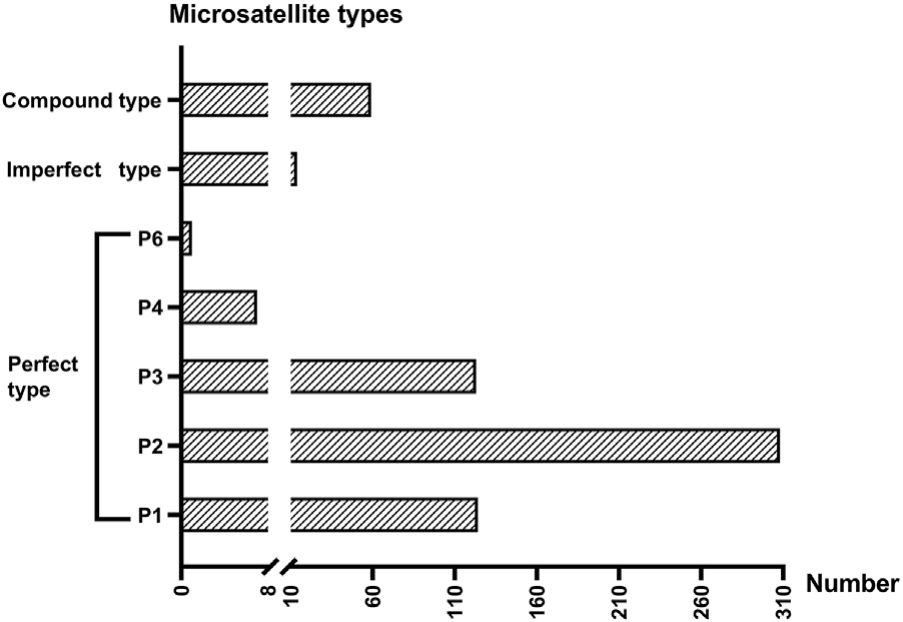
The number of three microsatellites types gained from six thelytokous *D. wani*. P1 ∼ P4 and P6 represent mono-, di-, tri-, tetra-, and hexanucleotide repeats of perfect types.

In total, 3,756,206 clean reads were obtained after low-quality sequences were removed. We then assembled these reads into 16,891 contigs and extracted the contigs containing microsatellite repeats. A total of 636 microsatellite-containing contigs were obtained from thelytokous *D*. *wani*. The numbers of perfect, compound, and imperfect microsatellites were 563, 59, and 14, respectively (Figure 5). The frequencies of each perfect microsatellite type were as follows: 19.50% (124 loci), 48.43% (308 loci), 19.34% (123 loci), 1.10% (7 loci), 0% (0 loci), and 0.16% (1 loci), respectively (Figure 5).

Ten primer pairs were designed and amplified (Table 3). Based on ten microsatellite loci, the genotyping error rate of the laboratory population was zero. The number of alleles at the 10 microsatellite loci ranged from 2 to 14. The observed heterozygosity (HO) ranged from 0.458 to 1, and the expected heterozygosity (HE) ranged from 0.503 to 0.884, indicating a high degree of heterozygosity in thelytokous *D*. *wani*. Most of the microsatellite loci were highly polymorphic (PIC >.5).

**TABLE 3:** 10 genetic markers isolated from thelytokous *D. wani*. The parameters are as follows: Number of alleles (NA), observed (HO) and expected (HE) heterozygosities, and the polymorphism information content (PIC).

Locus	Primer sequences (5′–3′)	Repeat motif	Tm (°C)	Size (bp)	NA	HO	HE	PIC	GenBank accession no.
DWTH217	F: GAT​ATG​CCA​ACA​CAG​CGT​GAT​TAT​CC	CTG 9	60	203	4	0.667	0.532	0.451	OP527026
	R: ATA​GTG​GTC​ATC​ACC​TTC​TTC​GC	CTG 9	60	203					
DWTH366	F: TGC​GAG​TTG​AGC​GTA​TAT​GCA​CAC	TG 12	60	147	7	0.952	0.863	0.822	OP527027
	R: TGT​TTG​CGA​TAT​ATA​CGC​GTA​TTG​C	TG 12	60	147					
DWTH119	F: ACA​CAC​AAC​ATT​TGC​GCG​C	CT 9	52	169	2	0.458	0.503	0.371	OP527031
	R: TGCGAGTTTCTCTCGGAC	CT 9	52	169					
DWTH75	F: TCC​ATT​TTC​ACT​GTA​TAG​AAC​GCG​T	TG 9	58	220	6	0.583	0.797	0.748	OP527028
	R: AGC​CAT​TAC​TCG​ACC​GTT​CAA​ACG​TGC​C	TG 9	58	220					
DWTH178	F: TCC​ATA​CAT​ATC​ATG​TTC​GTT​AGC​C	CT 11	58	200	8	0.875	0.801	0.753	OP527029
	R: AAG​ATT​TAT​ATT​CGT​ACC​AGC​GC	CT 11	58	200					
DWTH205	F: GAA​AGA​ATG​AGA​AAC​CAG​AAC	AG 21	54	130	14	0.864	0.884	0.851	OP527030
	R: AGA​TGA​CGT​GAT​TAT​ATC​GCG​TCC​GCA	AG 21	54	130					
DWTH151	F: AAG​CTG​TGA​GAG​TAG​GCA​TC	CT 13	52	240	9	1	0.876	0.841	OP527034
	R: GCG​ACG​TAA​TCA​GCG​CGT​TCA​C	CT 13	52	240					
DWTH67	F: TGC​CGG​TCA​AGA​GAG​ATA​GAC​ATC​GC	TC 11	52	234	5	0.714	0.761	0.703	OP527033
	R: TGA​CAT​GTT​CTC​ACT​GCA​GTA​GTA​GC	TC 11	52	234					
DWTH42	F: AGA​TAC​GCG​TAT​TCA​TAA​GCA​TTC	TC 15	52	262	8	0.708	0.791	0.742	OP527032
	R: ACA​CAT​TCC​TCG​TGT​CTT​CAC​GGC​AT	TC 15	52	262					
DWTH340	F: TGC​GGA​TAT​CAA​AGC​CGC​TA	GA 9	56	170	7	0.958	0.778	0.732	OP527035
	R: ACG​CTC​TCT​ATA​TGC​AAA​TAC​GAC	GA 9	56	170					

### 3.4 Microsatellite genotyping

The offsprings of thelytokous strain demonstrated the same heterozygous genotype as their parents based on four microsatellite loci (DWTH119, DWTH366, DWTH178, and DWTH340) (Table 4). Besides, random individuals in rearing laboratory colonies showed the same heterozygous genotypes and no individuals with homozygous genotypes were found. The results of allele segregation revealed that the observed rate of transition to homozygosity of a heterozygous locus was significantly different from that expected under automixis with gamete duplication, terminal fusion, or random fusion (Table 5). However, the observed rate was consistent with the values expected under automictic thelytoky with central fusion and apomixis (Table 5).

**TABLE 4:** Genotypes of parent, progeny, and random individuals of thelytokous *D. wani*. The numbers before and after the virgule indicate alleles in the species, where diploid females have two alleles and haploid males carry only one allele.

Locus	Laboratory colonies (parent and progeny)				Random laboratory colonies
	Parent (*n* = 8)	F1 (*n* = 96)	F2 (*n* = 63)	F3 (*n* = 60)	*n* = 96
DWTH119	169/171	169/171	169/171	169/171	169/171
DWTH366	145/147	145/147	145/147	145/147	145/147
DWTH178	191/197	191/197	191/197	191/197	191/197
DWTH 340	160/168	160/168	160/168	160/168	160/168

**TABLE 5:** Observed frequencies of heterozygous transition to homozygous loci for thelytokous *D. wani*, and agreement with theoretical frequencies under different cytological genetic mechanisms.

Locus	Nt	No	R (95%CI)	Automixy				Apomixis
				Gamete duplication (*r* = 1)	Terminal fusion (*r* = 1/3–1)	Central fusion (*r* = 0–1/3)	Random fusion (*r* = 1/3)	(*r* = 0)
DWTH119	219	0	0	***	***	NS	***	NS
DWTH366	219	0	0	***	***	NS	***	NS
DWTH178	219	0	0	***	***	NS	***	NS
DWTH340	219	0	0	***	***	NS	***	NS

Nt, number of heterozygous offspring; No, number of homozygous offspring; *R*, observed rate of transition to homozygosity; *r*, expected rate of transition to homozygosity (Pearcy et al., 2006; Pearcy et al., 2011b). Fisher’s exact test was used to test the consistency of *R* values with *r*.

NS indicates not significant and * * * indicates highly significant (*p* < .001). When *r* indicates a range, the test is performed by considering the *r* closest to *R* within the range.

## 4 Discussion

Chromosome fusion patterns are key to reveal sex determination in Hymenopteran species. Cytological observations showed that the thelytokous strain of *D*. *wani* did not undergo meiosis and did not produce polar bodies. Therefore, the thelytokous strain of *D*. *wani* did not undergo genetic recombination, and the cytological mechanism of diploidization in thelytokous *D*. *wani* was apomixis. Microsatellite data also supported the discovery of apomixis because no segregation of microsatellite loci occurred and the offspring maintained the same heterozygosity as their parents. Until now, apomictic thelytoky has only been reported in three parasitoids, *T*. *cacaeciae* (Vavre et al., 2004), *N*. *formosa* (Adachi-Hagimori et al., 2008), and *M. pulchricorni* (Tsutsui et al., 2014). Moreover, no bacterial endosymbionts caused thelytoky in *T*. *cacaeciae* or *M*. *pulchricornis*. In contrast, it was found that *Rickettsia* induced thelytoky in *N*. *formosa*. Thus, our results revealed that *D*. *wani* is the first eulophid parasitoid in Hymenoptera whose thelytoky was not induced by bacteria to form an apomictic thelytoky.

Compared with *T*. *cacaeciae*, *N*. *formosa*, and *M*. *pulchricornis*, some cytological characteristics of these three parasitoids differed from those of *D*. *wani*. Unlike *D*. *wani*, both thelytokous *N*. *formosa* and *T*. *cacaeciae* produced polar bodies during meiosis. Although *M*. *pulchricornis* and *D*. *wani* were similar in most aspects, minor differences existed in diploidization. 45 min after oviposition, the first anaphase of mitosis proceeded in thelytokous *D*. *wani*. In contrast, the first mitotic process in *M*. *pulchricornis* occurred slowly, beginning 1 h 50 min after egg oviposition (Tsutsui et al., 2014). The speed of embryonic cell division may be closely related to the growth and development of the species, especially during the egg stage. The egg period of thelytokous *M*. *pulchricornis* (2 days) was longer than that of thelytokous *D*. *wani* (1.1 days) at 25°C (Suzuki and Tanaka, 2007; Ye et al., 2018).

With the rapid development of genetic marker technology, an increasing number of studies have investigated cytological mechanisms using molecular methods (Adachi-Hagimori et al., 2008; Rey et al., 2011; Tsutsui et al., 2014; Alavi et al., 2018; Hellemans et al., 2019; Simonato et al., 2019; Smith et al., 2019; Tanaka and Daimon, 2019). Molecular methods allow for faster access to relevant mechanistic information than cytological observations (Adachi-Hagimori et al., 2008; Simonato et al., 2019). However, by ignoring cytological observations, it is impossible to determine whether the mechanism corresponds to actual processes (Adachi-Hagimori et al., 2008). Furthermore, it is difficult to investigate the differences in chromosomal segregation behaviour among different thelytokous species when no cytological observations are performed. Thus, we suggest that future research could involve a combination of cytology and molecular biology or genomics to explore the cytological mechanisms of thelytokous.

Moreover, the number of multilocus genotypes between thelytokous and arrhenotokous strains were applied recently to investigate apomixis (Wachi et al., 2021). Wachi et al. (2021) reported that thelytoky of *M*. *pulchricornis* was apomictic based on multilocus genotypes, which was consistent with previous cytological discoveries of Tsutsui et al. (2014). Additionally, the heterozygosity of field populations could be used to roughly infer cytological mechanisms. Simonato et al. (2019) suggested that thelytokous in field *Baryscapus servadeii* individuals infected *Rickettsia* likely corresponds to a mode of automictic thelytokous involving gamete duplication, which leads to full homozygosity of progeny. In contrast, field populations of the thelytokous strain of *D*. *wani* exhibit high heterozygosity, suggesting that the mechanism at play in *D. wani* is either automictic thelytoky involving central fusion or apomixis when only based on genetic markers. *Trichogramma cacaeciae*, which also has an apomictic thelytoky, displayed a higher frequency of heterozygosity in its field populations (Vavre et al., 2004). The reason for high heterozygosity may be due to the potential mechanism of Meselson effect (Mark Mark Welch and Meselson 2000), triploidization (Naito and Inomata, 2006), and hybridization (Vavre et al., 2004), which can accumulate new mutations. In contrast, for field *M*. *pulchricornis* population did not exhibit significantly higher heterozygosity than arrhenotokous strain probably due to their recent origin of thelytoky (Wachi et al., 2021). Therefore, high heterozygosity in field populations may be not always associated with apomixis due to different origin history of thelytoky. In the future research, exploring the origin of thelytoky in genome level is necessary to clear the heterozygosity of apomictic thelytoky.

The chromosomes of many parasitoids are predominantly metacentric and/or submetacentric (Gokhman, 2022). Our karyotyping of two strains of *D*. *wani* indicated diploid chromosome numbers of 2*n* = 10, consisting of four pairs of metacentric chromosomes and one pair of submetacentric chromosomes. At present, there are few karyotype studies on reproductive differentiation strains of conspecific Hymenoptera species. Gebiola et al. (2012) further investigated the thelytokous and arrhenotokous strains of the *P*. *soemius* which were a cryptic complex and demonstrated different chromosome morphologies (Gebiola et al., 2012). However, Gebiola et al. (2012) did not compare karyotypes between different strains in homogenous cryptic species. Thus, our study is the first to compare chromosome morphology among different strains of conspecific species of Hymenoptera parasitoids.

It is generally believed that thelytoky evolved from arrhenotoky in Hymenoptera (Cook 1993; Adachi-Hagimori et al., 2011; Ma et al., 2014; Sethuraman et al., 2022). A recent study suggested two independent origins for thelytokous *Dinocampus coccinellae* from the ancestral arrhenotokous strain (Sethuraman et al., 2022). However, in the process of evolution, thelytokous and arrhenotokous modes have reached different outcomes (Smith and Maynard-Smith, 1978). Arrhenotoky counterbalances this disadvantage by preventing the accumulation of deleterious mutations and creating new genetic recombination that may enhance adaptation (Hurst and Peck, 1996; Butlin 2002; Kraaijeveld et al., 2016). Genetic recombination is usually accomplished by homologous recombination of chromosomes during meiosis. In contrast, thelytoky accumulates increasing deleterious mutations and lacks long-term genetic flexibility offered by genetic variation and recombination, and may become extinct (Smith and Maynard-Smith, 1978). Therefore, arrhenotoky is widely regarded as the optimal reproductive mode for the long-term maintenance of species. In fact, the automictic thelytoky could undergo genetic recombination following completion of their normal first meiosis. However, apomictic thelytoky generally does not undergo meiosis. Thus, the accumulation effect of the deleterious variation generated by cytological mechanisms with automixis and apomixis is different. Apomictic thelytoky is thought to accumulate more deleterious variation than automictic thelytoky. Thus, more detailed studies are required to resolve the long-term maintenance of apomictic thelytokous species of *D*. *wani*.

## Data Availability

The original contributions presented in the study are publicly available. This data can be found here: https://www.ncbi.nlm.nih.gov/nuccore, accession numbers: OP527026 to OP527035.

## Supplementary material

Supplementary Table S1 The polymorphic markers developed for thelytokous Diglyphus wani.

## References

[B1] Adachi-Hagimori T. Miura K. Abe Y. (2011). Gene flow between sexual and asexual strains of parasitic wasps: A possible case of sympatric speciation caused by a parthenogenesis-inducing bacterium. J. Evol. Biol. 24 (6), 1254–1262. 10.1111/j.1420-9101.2011.02257.x 21457171

[B2] Adachi-Hagimori T. Miura K. Stouthamer R. (2008). A new cytogenetic mechanism for bacterial endosymbiont-induced parthenogenesis in Hymenoptera. Proc. R. Soc. Lond. B Biol. Sci. 275 (1652), 2667–2673. 10.1098/rspb.2008.0792 PMC2605818 18713719

[B3] Alavi Y. van Rooyen A. Elgar M. A. Jones T. M. Weeks A. R. (2018). Novel microsatellite markers suggest the mechanism of parthenogenesis in *Extatosoma tiaratum* is automixis with terminal fusion. Insect Sci. 25 (1), 24–32. 10.1111/1744-7917.12373 27345587

[B4] Archetti M. (2010). Complementation, genetic conflict, and the evolution of sex and recombination. J. Hered. 101, S21–S33. 10.1093/jhered/esq009 20200138

[B5] Belshaw R. Quicke D. L. (2003). The cytogenetics of thelytoky in a predominantly asexual parasitoid wasp with covert sex. Genome 46 (1), 170–173. 10.1139/g02-112 12669810

[B6] Beukeboom L. W. Pijnacker L. P. (2000). Automictic parthenogenesis in the parasitoid *Venturia canescens* (Hymenoptera: Ichneumonidae) revisited. Genome 43 (6), 939–944. 10.1139/g00-061 11195346

[B7] Butlin R. (2002). Evolution of sex: The costs and benefits of sex: New insights from old asexual lineages. Nat. Rev. Genet. 3 (4), 311–317. 10.1038/nrg749 11967555

[B8] Caron V. Norgate M. Ede F. J. Nyman T. Sunnucks P. (2013). Novel microsatellite dna markers indicate strict parthenogenesis and few genotypes in the invasive willow sawfly *nematus oligospilus* . Bull. Entomol. Res. 103 (1), 74–88. 10.1017/S0007485312000429 22929915

[B9] Clarke K. R. Gorley R. N. (2001). PRIMER version 5.0: User manual/tutorial. Plymouth: PRIMER-E Ltd.

[B10] Cook J. M. (1993). Sex determination in the Hymenoptera: A review of models and evidence. Heredity 71 (4), 421–435. 10.1038/hdy.1993.157

[B11] De Barro P. J. Driver F. (1997). Use of RAPD PCR to distinguish the B biotype from other biotypes of *Bemisia tabaci* (Gennadius) (Hemiptera: Aleyrodidae). Aust. J. Entomol. 36, 149–152. 10.1111/j.1440-6055.1997.tb01447.x

[B12] Dodds K. S. (1939). Oogenesis in *Neuroterus baccarum* L. Genetica 21, 177–190. 10.1007/BF01508151

[B13] Doncaster L. (1916). Gametogenesis and sex determination of the gall fly *Neuroterus lenticularis (Spathegaster baccarum*) III. Proc. Roy. Soc. Lond. B 89 (613), 183–200.

[B14] Du S. J. Ye F. Y. Wang Q. J. Liang Y. X. Wan W. J. Guo J. Y. (2022). Multiple data demonstrate that bacteria regulating reproduction could be not the cause for the thelytoky of *Diglyphus wani* (Hymenoptera: Eulophidae). Insects 13 (1), 9. 10.3390/insects13010009 PMC8777843 35055852

[B15] Du S. J. Yefremova Z. Ye F. Y. Zhu C. D. Guo J. Y. Liu W. X. (2021). Morphological and molecular identification of arrhenotokous strain of *Diglyphus wani* (Hymenoptera, Eulophidae) found in China as a control agent against agromyzid leafminers. Zookeys 1071, 109–126. 10.3897/zookeys.1071.72433 34887696 PMC8613133

[B16] Gebiola M. Giorgini M. Navone P. Bernardo U. (2012). A karyological study of the genus *pnigalio* schrank (Hymenoptera: Eulophidae): Assessing the taxonomic utility of chromosomes at the species level. Bull. Entomol. Res. 102 (1), 43–50. 10.1017/S0007485311000356 21736855

[B17] Giorgini M. Mancini D. Pedata P. A. (2007). “Cytological evidence for two different mechanisms of thelytokous parthenogenesis in *Encarsia* parasitoids harbouring *Wolbachia* or *Cardinium* bacteria. Poster,” in X European workshop on insect parasitoids, Erice, Italy, 17–21.

[B18] Gokhman V. E. (2022). Comparative karyotype analysis of parasitoid Hymenoptera (insecta): Major approaches, techniques, and results. Genes 13 (5), 751. 10.3390/genes13050751 35627136 PMC9141968

[B19] Gottlieb Y. Zchori-Fein E. (2001). Irreversible thelytokous reproduction in *Muscidifurax uniraptor* . Entomol. Exp. Appl. 100 (3), 271–278. 10.1046/j.1570-7458.2001.00874.x

[B20] Gottlieb Y. Zchori-Fein E. Werren J. H. Karr T. L. (2002). Diploidy restoration in *wolbachia*-infected *Muscidifurax uniraptor* (Hymenoptera: Pteromalidae). J. Invertebr. Pathol. 81 (3), 166–174. 10.1016/s0022-2011(02)00149-0 12507486

[B21] Heimpel G. E. de Boer J. G. (2008). Sex determination in the Hymenoptera. Annu. Rev. Entomol. 53, 209–230. 10.1146/annurev.ento.53.103106.093441 17803453

[B22] Hellemans S. Dolejsova K. Krivanek J. Fournier D. Hanus R. Roisin Y. (2019). Widespread occurrence of asexual reproduction in higher termites of the *Termes* group (Termitidae: Termitinae). BMC Evol. Biol. 19 (1), 131. 10.1186/s12862-019-1459-3 31226928 PMC6588926

[B23] Hurst L. D. Peck J. R. (1996). Recent advances in understanding of the evolution and maintenance of sex (vol 11, pg 46, 1996). Trends Ecol. Evol. 11 (7), 310. 21237760 10.1016/0169-5347(96)81041-x

[B24] Imai H. T. Crozier R. H. Taylor R. W. (1977). Karyotype evolution in Australian ants. Chromosoma 59 (4), 341–393. 10.1007/bf00327974

[B25] Kalinowski S. T. Taper M. L. Marshall T. C. (2007). Revising how the computer program CERVUS accommodates genotyping error increases success in paternity assignment. Mol. Ecol. 16 (5), 1099–1106. 10.1111/j.1365-294X.2007.03089.x 17305863

[B26] Kraaijeveld K. Anvar S. Y. Frank J. Schmitz A. Bast J. Wilbrandt J. (2016). Decay of sexual trait genes in an asexual parasitoid wasp. Genome Biol. Evol. 8 (12), 3685–3695. 10.1093/gbe/evw273 28172869 PMC5381511

[B27] Leniaud L. Darras H. Boulay R. Aron S. (2012). Social hybridogenesis in the clonal ant *Cataglyphis hispanica* . Curr. Biol. 22 (13), 1188–1193. 10.1016/j.cub.2012.04.060 22683263

[B28] Levan A. Fredga K. Sandberg A. A. (1964). Nomenclature for centromeric position on chromosomes. Hereditas 52, 201–220. 10.1111/j.1601-5223.1964.tb01953.x

[B29] Li Q. Wan J. M. (2005). SSRHunter: Development of a local searching software for SSR sites. Yi chuan 27 (5), 808–810. (In Chinese). 16257914

[B30] Luck R. F. Stouthamer R. Nunney L. P. (1993). “Sex determination and sex ratio patterns in parasitic Hymenoptera,” in Evolution and diversity of sex ratio in insects and mites (Wallingford, United Kingdom: CABI), 442–476.

[B31] Ma W. J. Pannebakker B. A. Beukeboom L. W. Schwander T. van de Zande L. (2014). Genetics of decayed sexual traits in a parasitoid wasp with endosymbiont-induced asexuality. Heredity 113 (5), 424–431. 10.1038/hdy.2014.43 24781809 PMC4220718

[B32] Ma W. J. Schwander T. (2017). Patterns and mechanisms in instances of endosymbiont-induced parthenogenesis. J. Evol. Biol. 30 (5), 868–888. 10.1111/jeb.13069 28299861

[B33] Mateo Leach I. M. Pannebakker B. A. Schneider M. V. Driessen G. Zande L. v. d. Beukeboom L. W. (2009). “Thelytoky in Hymenoptera with *Venturia canescens* and *Leptopilina clavipes* as case studies,” in Lost sex (Berlin, Germany: Springer), 347–375.

[B61] Mark Welch D. Meselson M. (2000). Evidence for the evolution of *bdelloid rotifers* without sexual reproduction or genetic exchange. Science 288 (5469), 1211–1215. 10.1126/science.288.5469.1211 10817991

[B35] Naito T. Inomata R. (2006). “A new triploid thelytokous species of the genus *Pachyprotasis* Hartig, 1837 (Hymenoptera: Tenthredinidae) from Japan and Korea,” in Recent sawfly research: Synthesis and prospects (Keltern: Goecke & Evers), 279–283.

[B36] Pannebakker B. A. Pijnacker L. P. Zwaan B. J. Beukeboom L. W. (2004). Cytology of *wolbachia*-induced parthenogenesis in *Leptopilina clavipes* (Hymenoptera: Figitidae). Genome 47 (2), 299–303. 10.1139/G03-137 15060582

[B37] Patwary M. U. Kenchington E. L. Bird C. J. Zouros E. (1994). The use of random amplified polymorphic DNA markers in genetic studies of the sea scallop *Placopecten magellanicus* (Gmelin, 1791). J. Shellfish Res. 13 (2), 547–553.

[B38] Peacock A. D. Sanderson A. R. (1939). The cytology of the thelytokous parthenogenetic sawfly *Thrinax maculata* . Trans. R. Soc. Edinb. 59 (3), 647–660. 10.1017/s0080456800017373

[B39] Pearcy M. Goodisman M. A. D. Keller L. (2011a). Sib mating without inbreeding in the longhorn crazy ant. Proc. R. Soc. Lond. Ser. B 278, 2677–2681. 10.1098/rspb.2010.2562 PMC3136830 21288949

[B40] Pearcy M. Hardy O. Aron S. (2006). Thelytokous parthenogenesis and its consequences on inbreeding in an ant. Heredity 96 (5), 377–382. 10.1038/sj.hdy.6800813 16552429

[B41] Pearcy M. Hardy O. J. Aron S. (2011b). Automictic parthenogenesis and rate of transition to homozygosity. Heredity 107 (2), 187–188. 10.1038/hdy.2010.172 21245891 PMC3178398

[B43] Rabeling C. Kronauer D. J. (2013). Thelytokous parthenogenesis in eusocial Hymenoptera. Annu. Rev. Entomol. 58, 273–292. 10.1146/annurev-ento-120811-153710 23072461

[B44] Rey O. Loiseau A. Facon B. Foucaud J. Orivel J. Cornuet J. M. (2011). Meiotic recombination dramatically decreased in thelytokous queens of the little fire ant and their sexually produced workers. Mol. Biol. Evol. 28 (9), 2591–2601. 10.1093/molbev/msr082 21459760

[B45] Sanderson A. R. (1988). Cytological investigations of parthenogenesis in gall wasps (Cynipidae, Hymenoptera). Genetica 77 (3), 189–216. 10.1007/BF00122389

[B46] Sethuraman A. Tovar A. Welch W. Dettmers R. Arce C. Skaggs T. (2022). Genome of the parasitoid wasp *Dinocampus coccinellae* reveals extensive duplications, accelerated evolution, and independent origins of thelytokous parthenogeny and solitary behavior. G3-Genes Genom. Genet. 12 (3), jkac001. 10.1093/g3journal/jkac001 PMC8896016 35100359

[B47] Simonato M. Pilati M. Magnoux E. Courtin C. Saune L. Rousselet J. (2019). A population genetic study of the egg parasitoid *Baryscapus servadeii* reveals large scale automictic parthenogenesis and almost fixed homozygosity. Biol. Control. 139, 104097. 10.1016/j.biocontrol.2019.104097

[B48] Smith J. M. Maynard-Smith J. (1978). The evolution of sex. Cambridge: Cambridge University Press.

[B49] Smith N. M. Wade C. Allsopp M. H. Harpur B. A. Zayed A. Rose S. A. (2019). Strikingly high levels of heterozygosity despite 20 years of inbreeding in a clonal honey bee. J. Evol. Biol. 32 (2), 144–152. 10.1111/jeb.13397 30414283

[B50] Stouthamer R. Kazmer D. J. (1994). Cytogenetics of microbe-associated parthenogenesis and its consequences for gene flow in *Trichogramma* wasps. Heredity 73 (3), 317–327. 10.1038/hdy.1994.139

[B51] Suomalainen E. Saura A. Lokki J. (1987). Cytology and evolution in parthenogenesis. Florida, United States: CRC Press.

[B52] Suzuki M. Tanaka T. (2007). Development of *Meteorus pulchricornis* and regulation of its noctuid host, *Pseudaletia separata* . J. Insect. Physiol. 53 (10), 1072–1078. 10.1016/j.jinsphys.2007.06.006 17675053

[B53] Tanaka M. Daimon T. (2019). First molecular genetic evidence for automictic parthenogenesis in cockroaches. Insect Sci. 26 (4), 649–655. 10.1111/1744-7917.12572 29389065

[B54] Thiel T. Michalek W. Varshney R. K. Graner A. (2003). Exploiting EST databases for the development and characterization of gene-derived SSR-markers in barley (*Hordeum vulgare* L.). Theor. Appl. Genet. 106 (3), 411–422. 10.1007/s00122-002-1031-0 12589540

[B55] Tsutsui Y. Maeto K. Hamaguchi K. Isaki Y. Takami Y. Naito T. (2014). Apomictic parthenogenesis in a parasitoid wasp *Meteorus pulchricornis*, uncommon in the haplodiploid order Hymenoptera. B. Entomol. Res. 104 (3), 307–313. 10.1017/s0007485314000017 24521569

[B56] van der Kooi C. J. Matthey-Doret C. Schwander T. (2017). Evolution and comparative ecology of parthenogenesis in haplodiploid arthropods. Evol. Lett. 1 (6), 304–316. 10.1002/evl3.30 30283658 PMC6121848

[B57] van Wilgenburg E. Driessen G. Beukeboom L. W. (2006). Single locus complementary sex determination in Hymenoptera: An "unintelligent" design? Front. Zool. 3, 1–15. 10.1186/1742-9994-3-1 16393347 PMC1360072

[B58] Vavre F. de Jong J. H. Stouthamer R. (2004). Cytogenetic mechanism and genetic consequences of thelytoky in the wasp *Trichogramma cacoeciae* . Heredity 93 (6), 592–596. 10.1038/sj.hdy.6800565 15329666

[B59] Wachi N. Gau J. J. Fujie S. Fukano K. Maeto K. (2021). Genomic population structure of sympatric sexual and asexual populations in a parasitic wasp, *Meteorus pulchricornis* (Hymenoptera: Braconidae), inferred from six hundred single-nucleotide polymorphism loci. Mol. Ecol. 30 (7), 1612–1623. 10.1111/mec.15834 33634920

[B60] Weber J. L. (1990). Informativeness of human (dC-dA)n (dG-dT)n polymorphisms. Genomics 7 (4), 524–530. 10.1016/0888-7543(90)90195-Z 1974878

[B62] Ye F. Y. Zhu C. D. Yefremova Z. Liu W. X. Guo J. Y. Wan F. H. (2018). Life history and biocontrol potential of the first female-producing parthenogenetic species of *Diglyphus* (Hymenoptera: Eulophidae) against agromyzid leafminers. Sci. Rep. 8, 3222. 10.1038/s41598-018-20972-3 29459647 PMC5818481

